# Urinary Biomarkers in the Assessment of Early Diabetic Nephropathy

**DOI:** 10.1155/2016/4626125

**Published:** 2016-06-16

**Authors:** Cristina Gluhovschi, Gheorghe Gluhovschi, Ligia Petrica, Romulus Timar, Silvia Velciov, Ioana Ionita, Adriana Kaycsa, Bogdan Timar

**Affiliations:** ^1^Division of Nephrology, University of Medicine and Pharmacy “V. Babes”, 300041 Timisoara, Romania; ^2^Romanian Academy of Medical Sciences, 300041 Timisoara, Romania; ^3^Department of Diabetes and Metabolic Diseases, University of Medicine and Pharmacy “V. Babes”, 300041 Timisoara, Romania; ^4^Division of Hematology, University of Medicine and Pharmacy “V. Babes”, 300041 Timisoara, Romania; ^5^Department of Biochemistry, University of Medicine and Pharmacy “V. Babes”, 300041 Timisoara, Romania

## Abstract

Diabetic nephropathy (DN) is a frequent and severe complication of diabetes mellitus (DM). Its diagnosis in incipient stages may allow prompt interventions and an improved prognosis. Towards this aim, biomarkers for detecting early DN can be used. Microalbuminuria has been proven a remarkably useful biomarker, being used for diagnosis of DN, for assessing its associated condition—mainly cardiovascular ones—and for monitoring its progression. New researches are pointing that some of these biomarkers (i.e., glomerular, tubular, inflammation markers, and biomarkers of oxidative stress) precede albuminuria in some patients. However, their usefulness is widely debated in the literature and has not yet led to the validation of a new “gold standard” biomarker for the early diagnosis of DN. Currently, microalbuminuria is an important biomarker for both glomerular and tubular injury. Other glomerular biomarkers (transferrin and ceruloplasmin) are under evaluation. Tubular biomarkers in DN seem to be of a paramount importance in the early diagnosis of DN since tubular lesions occur early. Additionally, biomarkers of inflammation, oxidative stress, podocyte biomarkers, and vascular biomarkers have been employed for assessing early DN. The purpose of this review is to provide an overview of the current biomarkers used for the diagnosis of early DN.

## 1. Introduction

Diabetic nephropathy (DN) represents an important cause of chronic kidney disease (CKD) that frequently leads to end stage renal disease (ESRD). Diabetes mellitus (DM) is a frequent disease and DN is one of its main complications. It is appreciated that up to 40% of the patients with type I and type II DM present DN [1]. In Western countries, diabetes is a leading cause of chronic kidney disease frequently leading to chronic renal replacement therapy (RRT) due to ESRD [2].

Taking into account the increased incidence of both DM and of DN, the detection of early DN is of paramount importance, in order to provide appropriate therapy that prevents or slows evolution towards ESRD.

Biomarkers play an important role in the early detection of DN. Among them, the best known is microalbuminuria. At the same time, microalbuminuria represents a marker of the generalized endothelial dysfunction present in DM, linking renal involvement with cardiovascular and cerebral impairment.

In time, it has been demonstrated that microalbuminuria reflects not only glomerular injury but also tubular lesions, filtered albumin being reabsorbed at tubular level. Additionally, new biomarkers have been studied in order to identify tubular lesions in DM.

The new tubular biomarkers have been detected in both type 1 and type 2 DM early renal dysfunction that precedes microalbuminuria. At present, the assessment of early DN involves numerous biomarkers. They span the period of normoalbuminuria that precedes microalbuminuria but also the evolution of renal involvement during microalbuminuria and macroalbuminuria.

Until they are universally accepted they are analyzed in relationship with the levels of albuminuria, especially of microalbuminuria.

At present, markers of inflammatory and oxidative processes accompanying DM and DN are also being assessed.

Since literature abounds in studies on markers highlighting renal dysfunction in different stages of the evolution of DM, we decided to restrict our study to the early phase of DN.

An update of the urinary biomarkers used in early DN is useful for establishing their role in the early diagnosis of this disease, with subsequent prophylactic and therapeutic implications. We insist on urinary biomarkers because they are easily drawn, which allows population screening, and because they can detect tubular lesions, which occur very early in DM.

Proteomics is an additional tool offering great prospects in DN assessment.

The origin of the biomarkers employed for assessing renal involvement in DM is diverse. Some of the biomarkers are constitutive elements of the nephron, such as markers at

- epithelial cell (podocyte) level, for example, nephrine and podocalyxin [3];
- glomerular basement membrane level: collagen and laminin [4];
- endothelial (VEGF) [5];
- tubular cell level, for example, NGAL, NAG, and KIM [6].

Some have mixed origin; they can originate both in tubular cells and in podocytes, for example, angiotensinogen [7, 8].

Some are derived from the circulation, for example, transferrin, ceruloplasmin, and immunoglobulins G and M. They pass into the urine because of glomerular lesions which result in increased permeability for plasma proteins.

There are several classifications addressing the diversity of urinary biomarkers in DM.

Matheson classifies the biomarkers according to both their origin and the pathologic processes impairing the nephron: kidney damage, oxidative stress, and inflammation:

- biomarkers of renal dysfunction,
- inflammatory biomarkers (cytokines and chemokines),
- oxidative stress biomarkers [9].

Another classification belongs to Hong and Chia who present 3 categories of biomarkers:

- glomerular,
- tubular,
- other proteins [10].

It should be noted that products of metabolism in DM are also eliminated in the urine, and they can trigger toxic effects, for example, advanced glycation end products (AGE).

Since we will frequently refer to microalbuminuria in presenting other biomarkers used in studying lesions of the nephron, namely, of the glomerulus and of the tubules, we will first present the main observations regarding microalbuminuria in diagnosing DN.

Recent literature uses new terms, like moderately increased albuminuria for microalbuminuria and severely increased albuminuria for macroalbuminuria. However, the classical terms of microalbuminuria and microalbuminuria continue to be in wide use, as they are more practical. This is why we will prefer them in the present paper.

Urinary biomarkers use in assessment of early diabetic nephropathy are presented in Table 1.

## 2. Microalbuminuria (Moderately Increased Albuminuria) in Type 1 DM

Microalbuminuria usually begins 5–10 years after the onset of type 1 DM [11].

Kidney biopsy examination in patients with type 1 DM and microalbuminuria most frequently finds normal histological aspects. However, DN lesions were found in a small number of patients [12].

According to McKenna and Thompson, microalbuminuria is predictive element of the future development of end stage renal disease [13].

Microalbuminuria can regress towards normoalbuminuria, it can persist as such, or it can progress towards albuminuria [14, 15].

The evolution of microalbuminuria towards macroalbuminuria is usually related to arterial hypertension and reduced GFR, an important part being generally played by risk factors [16].

Persistent microalbuminuria is related to future development of end stage renal disease and to cardiovascular risk [13].

It should be noted that diminution of GFR usually occurs after the development of microalbuminuria, but there are situations when even normoalbuminuria is accompanied by diminution of the GFR [17].

## 3. Microalbuminuria (Moderately Increased Albuminuria) in Type 2 DM

Microalbuminuria is an important biomarker in type 2 DM, being frequently used in population-based screenings.

Regarding the prevalence of microalbuminuria in type 2 DM patients we highlight a review of Newman et al. of 28 studies on 10,294 patients. They found microalbuminuria in 26% of the patients with ten-year duration of the disease [18].

A study on 24,000 patients found that Asian and Hispanic patients with type 2 DM present more often microalbuminuria (43%) than whites (33%) [19].

In China, Shanghai, microalbuminuria has a prevalence of 41% among patients with type 2 DM [20].

Hypertensive patients with type 2 DM present microalbuminuria more frequently [21].

Microalbuminuria can have a variable evolution. It can regress towards normal values, it can progress towards macroalbuminuria, or it can remain unchanged. In a study on 216 patients, Araki et al. found, after a 6-year follow-up, regression of microalbuminuria in 51% cases and progression to severely increased albuminuria in 28% cases [22].

The risk of progression to severely increased albuminuria is higher in patients with microalbuminuria as compared to patients with normoalbuminuria [18].

The diminution of GFR is also higher in patients with severely increased albuminuria than in those with microalbuminuria [23].

Glycemic control, ACE inhibitors, and ARBs for blood pressure control play an important role in the evolution of microalbuminuria. It should be mentioned that microalbuminuria has been considered a glomerular biomarker. To date, emerging data point to the role of the tubules in producing microalbuminuria [24, 25]. As such we did not include this marker among glomerular biomarkers but approached it separately, according to its potential role as both a glomerular and tubular biomarker.

### 3.1. Glomerular Biomarkers

#### 3.1.1. Urinary Transferrin

Transferrin is a protein with a molecular weight of 76.5 KDa. Because of its low molecular weight and its less ionic load it filters easily through the glomerular barrier [26].

As increased urinary transferrin was found in type 2 DM normoalbuminuric patients, concomitantly with urinary ceruloplasmin and immunoglobulin G, preceding microalbuminuria, it could be considered a biomarker of early DN [27].

In microalbuminuric patients the levels of urinary transferrin increase [28]. They also increase in patients with type 2 DM with vascular complications: coronary artery disease, diabetic retinopathy, and so forth [29].

Patients with initial high levels of urinary transferrin excretion will develop microalbuminuria more frequently than those with normal levels [30].

#### 3.1.2. Urinary Immunoglobulin G

It is an anionic plasma protein with a molecular weight of 150 KDa that crosses the glomerular barrier with difficulty [31].

As presented above, urinary IgG can be secreted before the stage of microalbuminuria, concomitantly with increased values of urinary transferrin, urinary ceruloplasmin, and urinary orosomucoid [27].

Increased elimination of urinary IgG could thus predict microalbuminuria in DM patients [27].

#### 3.1.3. Urinary Ceruloplasmin

Ceruloplasmin is a copper-transporting serum protein. It is filtered with difficulty at glomerular level because it is more negatively charged [32].

It was also found in some type 2 DM patients with normoalbuminuria, arguing in favour of its use for early detection of renal lesions, even prior to albuminuria: ceruloplasmin could have in type 2 DM patients a DN predictive effect, similar to urinary transferrin and urinary immunoglobulin G [27]. According to Yamazaki et al. the urinary ceruloplasmin excretion rate (CER) and clearance of ceruloplasmin increase in parallel with the progression of albuminuria [33].

In fact, in type 2 DM patients there could exist a parallelism between increased values of urinary transferrin, urinary immunoglobulin G, and urinary ceruloplasmin [27].

We conclude that urinary ceruloplasmin could be used for the early diagnosis of DN [34].

#### 3.1.4. Type IV Urinary Collagen

Type IV collagen is a component of the glomerular basement membrane and of the mesangial matrix [35].

In DN, lesions are produced both at glomerular capillary level and at mesangial level. Its excretion in urine might serve as early indicator of renal injury associated with DN [36].

Increased levels of type IV urinary collagen are reported for normoalbuminuric patients with type 1 DM. It could be a biomarker used for the early diagnosis of DN [37]. Other authors also report increased excretion of type IV collagen and of laminin in patients with type 1 DM [23].

High urinary type IV collagen excretion was also reported in normoalbuminuric patients with impaired glucose tolerance [38].

Urinary type IV collagen could reflect morphological renal alterations in patients with type 2 DM. A relationship between the severity of histological lesions and urinary type IV collagen was reported in patients with type 2 DM [39].

Type IV urinary collagen is considered to be a specific indicator of early DN [40].

It could also allow both detection of early DN in patients with type 2 DM and differential diagnosis with glomerulonephritis, where its levels are low [41].

#### 3.1.5. Urinary Laminin

Laminin is a component of the glomerular basement membrane. Its urinary excretion is increased in normoalbuminuric type 2 DM patients, being correlated with NAG (N-acetyl-beta-D-glucosaminidase) and alpha-1-microglobulin excretion. Concomitantly increased excretion of type IV collagen is found [4].

#### 3.1.6. Urinary Glycosaminoglycans

Glycosaminoglycans are components of the glomerular basement membrane as well as of the extracellular matrix. In DM alterations of these occur, the excretion of glycosaminoglycans being increased, even in normoalbuminuric patients [42].

Glycosaminoglycans are also present at the level of the tubular basement membrane. Urinary glycosaminoglycans are associated with other tubular biomarkers, for example, Tamm-Horsfall protein, which expresses a distal tubular dysfunction in diabetic patients [43].

#### 3.1.7. Lipocalin-Type Prostaglandin-D Synthase (L-PGDS)

It is a biomarker related to lesions of the glomerular capillary walls and reflects their increased permeability. It is mainly considered to predict renal lesions, being less relevant as an early marker of DN [44].

#### 3.1.8. Urinary IgM and Urinary Fibronectin

These were studied only sporadically, without sufficient data to support their use as markers of early DN.

Urinary fibronectin excretion is significantly increased in DM patients only if they present microalbuminuria [45]. IgM is an indicator of impaired kidney function [46].

Although the use of urinary glomerular biomarkers has not become current practice yet, glomerular biomarkers have been reported in some normoalbuminuric patients, leading to the conclusion that albuminuria might not represent the most sensitive glomerular biomarker. However, their clinical applicability needs to be confirmed in high-quality validation studies [31].

### 3.2. Tubular Biomarkers

DN is manifested mainly by well-known glomerular lesions. The aforementioned biomarkers are identified already precociously early in early DN. Tubulointerstitial lesions are associated with glomerular injury during DN [47]. Tubular biomarkers have shown that tubular dysfunction can be present early in DN, sometimes preceding glomerular injury. These biomarkers have proven highly sensitive as compared to microalbuminuria, which is considered the gold standard biomarker of DN. In fact, presently, microalbuminuria is regarded not only as a glomerular biomarker, but also as a tubular one.

#### 3.2.1. Neutrophil Gelatinase-Associated Lipocalin (NGAL)

NGAL—neutrophil gelatinase-associated lipocalin—is a glycoprotein present in the kidneys at tubular cell level and is considered to be protective against renal damage [48].

Urinary NGAL is a biomarker used in assessing tubular lesions in DM, its increased values being present even in the initial phases of the disease, namely, in normoalbuminuric patients [49].

Thus, in type 1 DM high urinary NGAL can precede microalbuminuria [50, 51].

Urinary NGAL had high values in type 2 DM patients with normoalbuminuria, increasing progressively in patients with microalbuminuria and macroalbuminuria. The values of KIM-1 (kidney injury molecule-1) increased in parallel, indicating an early and progressive lesion [52].

However, Fu et al. reported in type 2 DM patients who present hyperfiltration and increased values of urinary NGAL, as well as of urinary KIM-1, as compared to the values of patients with normal GFR [53].

Urinary NGAL shows the precocity of tubular lesions in patients with prediabetes [54].

Urinary NGAL in type 2 DM patients could have a role in predicting the evolution of disease [55].

#### 3.2.2. Urinary Alpha-1-Microglobulin

Urinary alpha-1-microglobulin is a serum protein with low molecular weight (27-kDa), which allows it to get easily filtered through the glomerular capillary wall. Once it arrives in the proximal tubule, alpha-1-microglobulin is reabsorbed and metabolized. Tubular dysfunction leads to alteration of reabsorption with increased excretion in the urine [56].

In a cross-sectional study, Hong et al. analyzed 590 type 2 DM patients and found that 33.6% patients with normoalbuminuria presented increased values of urinary alpha-1-microglobulin, a fact that could be explained by tubular injury that precedes the occurrence of microalbuminuria, being a more sensitive and an earlier urinary biomarker [57]. However, alpha-1-microalbuminuria can be absent in some patients with albuminuria [57]. This is why assessments of alpha-1-microglobulin are associated with the assessment of other urinary biomarkers, urinary albumin included.

Petrica et al. reported high values of urinary alpha-1-microglobulin in normoalbuminuric patients, a fact pleading for an early tubular injury in type 2 DM in this stage. They did not find correlations between urinary alpha-1-microglobulin, beta-2 microglobulin, and the albumin/creatinine ratio with plasma asymmetric dimethyl-arginine. This could plead for dissociation between tubular and endothelial dysfunction [58].

Alpha-1-microglobulin in early stages of DM could also have a role in predicting DN [59]. It is in fact an inexpensive biomarker of early diagnosis of DN [60].

#### 3.2.3. Urinary KIM-1 (Kidney Injury Molecule-1)

KIM-1 is a transmembrane glycoprotein located at the level of the proximal tubular cells. It is eliminated in urine in case of injury at this level. It is a sensitive biomarker used with good results in acute kidney injury [61].

Petrica et al. reported in normoalbuminuric type 2 DM patients high values of urinary KIM-1, which indicates lesions of the proximal tubule in early stages of the disease. Patients with microalbuminuria have higher urinary KIM-1 values than those with normoalbuminuria [62].

de Carvalho et al. reported in type 2 DM normoalbuminuric patients high values of KIM-1, these values increasing progressively in patients with microalbuminuria and macroalbuminuria. NGAL values studied concomitantly presented similar evolutions [52].

Moreover, KIM-1 presents higher elimination in type 2 DM patients with hyperfiltration than in patients with normal glomerular filtration. NGAL has a similar evolution. These biomarkers—KIM-1 and NGAL—could plead for a deleterious lesional effect of hyperfiltration on the proximal tubule [53].

Nielsen et al. however could not demonstrate a value of urinary KIM-1 that could be predictive of the evolution of glomerular function (GFR) in patients with type 1 DM [63].

According to Nielsen et al. it has no prognostic utility in type 2 DM patients either [64].

#### 3.2.4. Urinary N-Acetyl-*β*-D glucosaminidase (NAG)

NAG is an enzyme located in the lysosomes of proximal tubular cells [65].

In case of dysfunction, namely, of injury of proximal tubular cells, NAG is eliminated into the urine in higher quantities, being a sensitive tubular biomarker. This can precede the appearance of microalbuminuria in type 1 DM [66].

Elevated serum Cys C levels and urinary NAG activities were found only in normoalbuminurics, not in controls. In addition, elevated urinary ALP and LDH activities were also found in microalbuminurics [67].

Other authors, like Ambade et al., did not find that urinary NAG has clinical significance as an early biomarker of DN [68].

In type 2 DM urinary NAG excretion increases proportionally to the duration of diabetes. It occurs much earlier than albuminuria. NAG can be considered an early tubular biomarker [69].

Assal et al. consider that urinary NAG is the most sensitive biomarker for detecting early damage in diabetic patients [70].

#### 3.2.5. Urinary Angiotensinogen

The renin angiotensin aldosterone system (RAAS) is involved in the pathogenesis of DN. The constitutive elements of RAAS are present at kidney level, defining a local RAAS.

Urinary angiotensinogen can represent a biomarker for the activation of RAAS in DM [71].

High urinary angiotensinogen precedes in type 1 DM patients the occurrence of microalbuminuria [72]. This could have a predictive role in normotensive type 1 DM patients [73].

Urinary angiotensinogen in normoalbuminuric type 2 DM patients is higher than in controls and it increases progressively in microalbuminuric and especially in macroalbuminuric patients [73].

Urinary angiotensinogen can be considered an early biomarker of DN [72].

In type 2 DM patients, urinary angiotensinogen is correlated with alpha-1-microglobulin [8].

Kim et al. did not confirm these observations in a study on type 2 DM patients. They found that the values of urinary angiotensinogen are not different from those of the controls, in normoalbuminuric and microalbuminuric type 2 DM patients, but higher values were described in macroalbuminuric patients [74].

These observations point to the need of further studies necessary for the validation of this biomarker.

Increased urinary angiotensinogen could represent a risk factor in renal and cardiovascular complications [75].

Since activation of RAAS could intervene in the evolution of DN, administration of ACE-I is recommended.

At the same time, urinary angiotensinogen could be a marker for assessing the renoprotective effects of alogliptin to type 2 DM patients [76].

#### 3.2.6. Cystatin C

It is a low molecular weight protein having the role of cysteine protease. Cystatin is produced by the nucleated cells in the body [77].

It is filtered at glomerular level and is reabsorbed in the tubules. Cystatin is used for evaluating renal function. Assessment of GFR by means of cystatin C is considered to be a method that is not influenced by body mass, being comparable and even better than methods using serum creatinine [78].

Serum cystatin is also considered a sensitive biomarker as it detects minor glomerular injury [79].

Urinary cystatin C indicates tubular injury. It increases early in diabetes and prediabetic nephropathy [80].

Patients with microalbuminuria present higher values of urinary cystatin C than those without microalbuminuria, urinary cystatin C having a predictive role for the progression of diabetic nephropathy (DN) [81].

Urinary cystatin C level could be an independent factor for identifying renal dysfunction in type 2 DM patients with normoalbuminuria, including patients with GFR <60/mL/min/1.73 m^2^ [77]. Uslu et al. find a significant positive correlation between serum cystatin C, urinary NAG, lacticodehydrogenase, alkaline phosphatase activities, and serum creatinine levels [67].

Serum and urinary cystatin C are useful biomarkers for assessing early nephropathy in type 2 DM [77].

#### 3.2.7. L-FABP (Liver-Type Fatty Acid Binding Protein)

Urinary L-FABP is a protein with low molecular weight expressed in the cytoplasm of human proximal tubular cells [82]. It is also expressed at liver level.

Increased urinary L-FABP was found in type 1 DM patients who presented normoalbuminuria, having a predictive role regarding the progression towards microalbuminuria and of microalbuminuria towards macroalbuminuria [83].

Patients with type 2 DM with normoalbuminuria also presented high levels of urinary L-FABP, this protein being considered as a useful biomarker for diagnosing early diabetic nephropathy. In fact, urinary L-FABP has been confirmed as a tubular biomarker by the Ministry of Health and Welfare in Japan [82].

The L-FABP factor is also related to the severity of DN. The values of urinary L-FABP increase with the decline of renal function [84].

Although some authors, like Chou et al., do not ascribe a predictive role to urinary L-FABP in type 2 DM patients [85], others, like Panduru et al., consider that urinary L-FABP is an independent predictor of the progression of DN [86].

#### 3.2.8. Nephrinuria

Nephrine is a transmembrane protein in the structure of the slit diaphragm [87].

In DM podocyte dysfunction is present. DN is considered a podocytopathy [88]. Injury of the slit diaphragm leads to nephrinuria.

Nephrinuria can occur in some type 1 DM patients prior to microalbuminuria [89]. Nephrinuria was also reported in some normoalbuminuric type 2 DM patients [62, 90].

Nephrinuria is related to podocyte injury representing a biomarker of early glomerular injury [91].

Dysregulation of nephrine in podocytes in DN could lead to nephrinuria in normoalbuminuric patients, preceding microalbuminuria [92].

In albuminuric patients, nephrinuria is positively correlated with albuminuria and negatively correlated with GFR, being a biomarker of DN in other phases of DM as well.

Podocyte impairment in DM involves not only nephrine but also other podocyte elements, for example, VEGF. Thus, in normoalbuminuric DM patients nephrinuria is correlated with urinary elimination of VEGF [62].

Tubular biomarkers seem to play an important role in the early diagnosis of DN. They manage to show, in most cases, that microalbuminuria does not represent a reliable biomarker for diagnosing incipient lesions of DN. However, up to now, none of these biomarkers has been established as gold standard for the identification of early DN.

### 3.3. Markers of Inflammation

DM is accompanied by chronic inflammatory processes affecting the whole body, the kidneys included. Mediators of inflammation, like cytokines and chemokines, are present in these processes. Some of them are useful as markers of inflammation.

#### 3.3.1. Tumour Necrosis Factor Alpha (TNF Alpha)

Urinary TNF alpha presents in type 2 DM patients with microalbuminuria and macroalbuminuria higher values than in patients with normoalbuminuria. Urinary TNF alpha is correlated with urinary NAG, a marker of tubular lesions [93].

Cherney et al. analyzed in a complex study on normoalbuminuric type 1 DM patients forty-two urinary cytokines/chemokines. They found that the urinary level of IL6 and IL8, the platelet-derived growth factor, and RANTES are not altered in patients with normal albumin-creatinine ratio.

Higher urinary excretion of these markers is associated with microalbuminuria. Cherney et al. consider that these markers could have a role in assessing the risk of DN in patients with type 1 DM [94].

In type 1 DM patients, renal hyperfiltration is related to increased excretion of inflammatory cytokines/chemokines [95].

Tashiro et al. found in type 2 DM patients that IL8 is high in early stages of DN and MCP-1 increases in advanced stages [96].

A study on type 2 DM patients with normoalbuminuria and microalbuminuria found higher values of IL8, IP10, MCP-1, G-CSF, EOTAXIN, and RANTES in patients with microalbuminuria than in normoalbuminurics or in controls. Their assessment would be useful in the early diagnosis and treatment of DN [97].

Ibrahim and Rached also found that urinary MCP-1 is higher in patients with microalbuminuria than in normoalbuminurics or healthy controls [98].

#### 3.3.2. Urinary Orosomucoid

Orosomucoid represents a glycoprotein involved in inflammatory processes.

Urinary orosomucoid has higher values in type 1 DM patients with normoalbuminuria than in controls. These values increase in patients with microalbuminuria and macroalbuminuria [99]. Type 2 DM patients presented increased excretion of orosomucoid in the urine, in parallel with the excretion of immunoglobulin G, ceruloplasmin, and transferrin [16]. El-Beblawy et al. appreciate that orosomucoid is a significant independent factor for diabetic microvascular complications and can be considered as an early marker of renal injury [100].

Urinary orosomucoid excretion rate in type 2 DM patients predicts cardiovascular mortality [101].

Urinary markers of inflammation are useful for assessing inflammatory processes in DN, even in early stages.

### 3.4. Oxidative Stress Biomarkers

Oxidative stress plays an important part in the development and progression of DN [102].

#### 3.4.1. Urinary 8-Oxo-7,8-dihydro-2-deoxyguanosine (8-oHdG)

8-oHdG is produced secondary to oxidative DNA damage. It is eliminated into the urine without being metabolized [103]. At present, it is considered a marker for oxidative stress.

After a 5-year follow-up, Hinokio et al. find that 8-oxodG in urine is a useful clinical marker to predict the development of diabetic nephropathy in diabetic patients. There was a significant progression of diabetic nephropathy in the patients with higher excretion of 8-oxodG in urine compared with the patients with moderate or lower excretion of 8-oxodG [104].

Leinonen et al. reported increased excretion of 8-oHdG in type 1 DM patients 9 years after the onset of disease, mainly related to poor glycemic control [105].

The urinary 8-oHdG marker of oxidation would be, according to Broedbaek et al., a predictor of long-term mortality in DM [106].

#### 3.4.2. Heart Fatty Acid Binding Protein (H-FABP)

Heart fatty acid binding protein (H-FABP**)** is a marker of distal tubular damage.

In a study on a cohort of type 1 and type 2 DM patients and an assessment of their markers of glomerular lesions (IgG), markers of proximal tubular lesions (urinary KIM-1, NAG, NGAL, and cystatin), and a marker of distal tubular lesions (urinary H-FABP) in relationship with albuminuria and GFR, Nauta et al. reported higher values of urinary NAG, NGAL, and H-FABP in normoalbuminurics than in controls. On the other hand, the values of urinary cystatin C were low [107].

This shows that normoalbuminuric DM patients present both proximal and distal tubular lesions.

#### 3.4.3. Urinary Advanced Glycation End Products (AGE)

AGE eliminated in the urine induce a toxic tubular effect producing tubular dysfunction.

In type 2 DM patients with normoalbuminuria high values of urinary alpha-1-microglobulin and of urinary KIM-1 were found secondary to tubular dysfunction prior to the onset of microalbuminuria. At the same time, urinary AGE were high, being correlated with these markers [108].

Turk et al. found in type 2 DM patients high values of urinary AGE in 50% of the patients with normoalbuminuria and in 85% of those with microalbuminuria [109].

Pentosidine, a component of AGE, is a biomarker for their formation and accumulation [110].

Piarulli et al. found in patients with microalbuminuria higher values of pentosidine than in patients with normoalbuminuria [111].

#### 3.4.4. Podocytes

Podocyte lesions appear during DM and DN, respectively, the disease being considered a podocytopathy as mentioned above.

The assessment of podocyte injury can be accomplished by monitoring the number of podocyte cells in the urine or, more precisely, by means of using podocyte urinary biomarkers (podocalyxin and nephrine).

A study on DM patients found that the values of the number of urinary podocytes in normoalbuminuric patients are not significantly different from those of controls. In patients with microalbuminuria and nephrotic syndrome, the number of urinary podocytes is higher. It is correlated with urinary osteopontin and urinary IgM [33].

Urinary podocalyxin originates in the podocyte apical surface, occurring in vesicle form. In DM patients, the podocalyxin level presented higher levels in patients with microalbuminuria than in patients with normoalbuminuria [112].

Another study on DM patients found high values of urinary podocalyxin in more than half of the patients with normoalbuminuria, these values being higher in patients with microalbuminuria and macroalbuminuria.

Urinary podocalyxin is correlated with the values of urinary NAG and of urinary beta 2 microglobulin [113].

Hara et al. consider that urinary podocalyxin can be an early biomarker for detecting early podocyte injury in patients with DM.

Zheng assessed the urinary microRNA profile of podocyte-associated molecules (synaptopodin, podocalyxin, CD2-AP, *α*-actin4, and podocin) as biomarkers in patients with normoalbuminuria, microalbuminuria, and macroalbuminuria and they reported its increase during the progression of DN [114].

#### 3.4.5. Vascular Endothelial Growth Factor (VEGF)

VEGF is a proangiogenic factor produced mainly by the podocytes at nephron level. Urinary VEGF can be considered a podocyte biomarker.

Urinary VEGF was detected in type 2 DM patients, being correlated in these patients with urinary alpha-1-microglobulin, a biomarker for proximal tubular lesions [62].

Kim et al. found that VEGF is excreted at higher values than controls in normoalbuminuric type 2 DM patients. The values increase in patients with microalbuminuria and macroalbuminuria [115].

Fetuin A is glycosylated glycoprotein was considered an inhibitor for ectopic calcium deposition and promoter of insulin resistance. Fetuin A inhibits the calcification of atherosclerotic plaques in diabetes mellitus [116]. It was found that elevated urinary Fetuin A excretion is a risk for development of diabetic nephropathy [117].

### 3.5. Other Urinary Biomarkers Used in Evaluating Early DN

Numerous urinary markers have been suggested for assessing early DN. Some of them have been introduced in use only recently.

Urinary retinol-binding protein is a low molecular weight protein that was found to have high urinary values (together with NAG) in normoalbuminuric patients, reflecting tubular dysfunction in early DN [118].

The value of serum retinol-binding protein 4 as a biomarker in assessing the severity of coronary artery disease is to be mentioned [119].

Urinary retinol-binding protein 4 as a biomarker in assessing DN needs further studies.

Urinary vitamin D binding protein can plays the role as biomarker. In type 2 DM it is attributed a potential role in early diagnosis of DN [120].

Urinary heme oxygenase-1 was found in type 2 DM patients before the onset of significant albuminuria, thus being a possible biomarker of early DN [121]. In fact oxidative stress activation is expected in DN.

Periostin is a cell adhesion molecule which is not normally present in kidneys. In tubulointerstitial lesions it is however expressed in the kidneys, being eliminated in the urine. This is why urinary periostin could be used as a marker of injury at this level.

Since high levels of periostin can be identified in DM patients before significant albuminuria, periostin could represent a marker of diabetic renal injury [122].

Urinary alpha klotho presents higher values in normoalbuminuric type 2 DM patients than in controls. It can also be a marker of diabetic injury [123].

Analyzing a group of normoalbuminuric, microalbuminuric, and macroalbuminuric type 2 DM patients, Sun et al. noted that the urinary level of microvesicle-bound dipeptidyl peptidase-IV is related to the severity of DN [124].

Recent studies point to the usage of urine-specific microRNA as a biomarker for early stages of DN. Analyzing the studies in the literature, Yang et al. issued the hypothesis that urine-specific microRNA would be a marker that can be used in the early stages of DN [125].

Recently, Argyropoulos highlighted the predictive role of urinary microRNA regarding microalbuminuria in type 1 DM [126].

Adipokine zinc-alpha-2 glycoprotein is assigned to the major histocompatibility complex class I of proteins [127].

Urinary adipokine zinc-alpha-2 glycoprotein is present earlier than microalbuminuria in diabetic nephropathy. It could be a useful biomarker for diagnosing early DN [128]. Lim et al. also appreciate adipokine zinc-alpha-2 glycoprotein as a novel urinary biomarker for normoalbuminuric diabetic nephropathy [129].

#### 3.5.1. Proteomics

At present proteomic investigations are engaged in identification of new urinary biomarkers to be used in the early diagnosis of DN.

In fact, proteomics studies noted the fact that microalbuminuria is not a perfect biomarker for early detection of DN [130, 131].

Urinary proteomics begins to stand out as a noninvasive method of detecting early DN.

Among proteomics studies on diagnosing DN we can mention those of Zürbig et al., who reported that collagen fragments were a prominent biomarker 3–5 years before the onset of microalbuminuria [132].

A potential role is also attributed to exosome proteomics for identifying new biomarkers for DN [133]. Zubiri et al. showed a panel of 3 proteins which is differentially present in urinary exosomes from DN patients [134]. Urinary proteomic analysis can have an important role in the implementation of new biomarkers in DN [135].

At present, the prospect of discovering new biomarkers in DM and DN respectively is incumbent both on proteomics and on genomics, transcriptomics, and metabolomics [136].

## 4. Conclusions

Urinary biomarkers allow an assessment of early DN.

Microalbuminuria, although frequently contested as a biomarker of early DN, is used so far as reference biomarker in assessing other urinary biomarkers in early DN. Until present there is no other biomarker that can substitute in practice microalbuminuria, the new biomarkers being sustained by limited studies and requiring validation.

The concomitant assessment of several urinary biomarkers in relationship with microalbuminuria could represent at present a method of diagnosing early DN. The great progress in discovering new biomarkers could lead to the development of an “ideal” urinary biomarker to detect early diabetic DN in the future.

Progresses in the field of urinary biomarkers in DN, promising both in proteomics and in other modern techniques, develop remarkably at present.

## Figures and Tables

**Table 1:** Urinary biomarkers in the assessment of early diabetic nephropathy.

Glomerular biomarkers		Tubular biomarkers
Transferrin	*Microalbuminuria* The main marker in current use	Neutrophil gelatinase-associated lipocalin (NGAL)
Immunoglobulin G		Alpha-1-microglobulin
Ceruloplasmin		Kidney injury molecule-1 (KIM-1)
Type IV collagen		N-acetyl-*β*-D glucosaminidase
Laminin		Angiotensinogen
Glycosaminoglycans		Cystatin C
Lipocalin-type prostaglandin D synthase		Liver-type fatty acid binding protein
Fibronectin		Nephrine
Podocytes-podocalyxin		Heart fatty binding protein
Vascular endothelial growth factor/VEGF		Advanced glycation end products

Inflammatory biomarkers	Other new markers under study	Oxidative stress biomarkers

Tumor necrosis factor alpha	Retinol binding protein-4	8-Oxo-7,8-dihydro-2′-deoxyguanosine
Orosomucoid	Vitamin D binding protein	
	Heme oxygenase-1	
	Periostin	
	Alpha klotho	
	Microvesicle-bound dipeptidyl peptidase IV	
	MicroRNA	
	Adipokinesine alpha-2 glycoprotein	
